# Health insurance coverages and demand for modern contraceptive choices among reproductive-age women in Thailand

**DOI:** 10.1186/s13561-026-00738-8

**Published:** 2026-02-06

**Authors:** Nattanon Phonkacha, Piriya Pholphirul

**Affiliations:** Graduate School of Development Economics, National Institute of Development Administration, Serithai Road, Klong-Chan, Bangkapi, Bangkok, 10240 Thailand

**Keywords:** Modern contraception, Contraceptive choice, Health insurance coverage, Reproductive-age women, Thailand

## Abstract

Contraception is crucial for women's well-being and national development, which is particularly salient in Thailand's rapidly aging society where maximizing the health of the working-age population is essential. While health insurance is pivotal, this research moves beyond analyzing financial barriers to examine the distinct structural influence of Thailand's three major health insurance systems—the Universal Coverage Scheme (UCS), Social Security Scheme (SSS), and Civil Servant Medical Benefit Scheme (CSMBS)—on modern contraceptive method choices among reproductive-age women. Using data from the 2022 Multiple Indicator Cluster Survey (MICS) (*n* = 10,922 women currently using contraception), a Multinomial Logistic Regression model yielded significant findings. The results demonstrate that the specific scheme structure, rather than just the presence of coverage, significantly predicts method selection. Women under the Social Security Scheme (SSS) showed a statistically significant higher propensity for hormonal methods (pills, injections, implants). In contrast, members of the Civil Servant Medical Benefit Scheme (CSMBS) showed a significant preference for device-based methods (condoms, IUDs, diaphragms). These distinct preferences align with the structural incentives of the schemes: the SSS's capitation payment model may favor time-efficient hormonal dispensing, while the CSMBS's fee-for-service model and provider flexibility facilitate access to medical devices and non-hormonal options. Additionally, religious beliefs and concerns about hormonal side effects (observed among highly educated, affluent women) played a substantial role in the adoption of natural methods. This study underscores the need for public health policies that move beyond guaranteeing financial access and consider the structural diversity of health insurance systems to promote appropriate and sustainable contraceptive access and informed choice.

## Introduction

Contraception is fundamentally important for individual health and societal progress, serving as a core component of reproductive rights. It empowers women to make autonomous decisions about their bodies and family size, enabling them to plan pregnancies in alignment with their physical, mental, social, and economic readiness. This capability is critical for reducing unintended pregnancies, unsafe abortions, and adverse maternal and infant mortality rates [22],Guttmacher [11]. Beyond risk prevention, effective family planning improves maternal and child health by facilitating appropriate birth spacing, thereby fostering better long-term health outcomes for the entire family [27].

Access to effective modern family planning services is thus crucial for promoting women's overall health and socioeconomic development. Individually, family planning enhances women's educational and career opportunities, leading to increased participation in economic activities and contributing to the realization of their full potential [17]. This positive impact on women's autonomy and economic activity is essential for long-term national development, which is particularly vital in demographic contexts like Thailand's, where maximizing the health and productivity of the working-age population is paramount.

Historically, contraception has evolved dramatically. Modern methods leverage advanced scientific principles and technology—including pills, injections, implants, Intrauterine Devices (IUDs), and sterilization—offering high efficacy and a diverse range of choices. These scientifically validated methods are proven safe and highly effective when used correctly. In contrast, traditional methods (e.g., the rhythm method, coitus interruptus) typically exhibit very low efficacy, carry a high risk of failure, and can sometimes pose health hazards. Global data from 2021 reveal that 77% of women seeking to avoid pregnancy primarily use modern contraceptive methods, reflecting not only improved access but also women's growing autonomy in shaping their futures [18].

Over the past decade, modern contraception has gained increasing prominence. Data from the United Nations [24] reveal that 77% of women seeking to avoid pregnancy primarily used modern contraceptive methods in 2021. Globally, the number of women using modern contraception nearly doubled from 467 million in 1990 to 874 million in 2021 [22]. This surge not only reflects improved access but also signifies women's growing autonomy in shaping their futures, leading to substantial social and economic transformations [24].

At the macro level, the increased utilization of modern contraception aligns closely with achieving the Sustainable Development Goals (SDGs), specifically Goal 3 (Good Health and Well-being) and Goal 5 (Gender Equality) [23]. While analyzing contraceptive type reveals varying regional demands, modern methods consistently remain more popular than traditional ones worldwide. Despite this global rise, contraceptive utilization in individual countries remains significantly influenced by socioeconomic disparities, educational attainment, geographical location, and sociocultural factors, presenting numerous challenges to access [5, 10, 13]. Furthermore, variations in public policies and health infrastructure play a crucial role in determining the supply and demand for family planning services [4].

In public health economics, health insurance systems are crucial for promoting access to health services by mitigating financial barriers [6, 19]. Studies confirm that general health insurance coverage positively correlates with overall contraceptive uptake [3, 14]. However, focusing only on whether a woman is insured overlooks a critical nuance: the mechanism of coverage. When insurance systems are structurally diverse, they can influence not just whether a woman uses contraception, but which specific method she selects. Therefore, systematically studying the relationship between health insurance type and the demand for specific modern contraceptive methods is essential for understanding the health system's mechanisms and their impact on women's decisions (e.g., [3, 14]).

Thailand offers a compelling case study for analyzing the dynamics of modern contraceptive demand. Propelled by significant economic and social advancements over several decades, Thailand has consistently invested in its public health system and family planning programs, resulting in a substantial decrease in the Total Fertility Rate [29]. This study focuses on Thailand’s unique health financing landscape, which comprises three distinct, state-mandated schemes: the Universal Coverage Scheme (UCS), the Social Security Scheme (SSS), and the Civil Servant Medical Benefit Scheme (CSMBS). While all three schemes guarantee basic family planning, they fundamentally differ in their financing, provider payment mechanisms, and service delivery networks [16].

Therefore, this study is guided by the core research question of why a woman's specific health insurance scheme might affect her choice of contraceptive method (e.g., sterilization versus oral pills), even when controlling for her age, income, and education. We hypothesize that these scheme differences create specific incentives and barriers through three mechanisms:

- *Differential Financial Burden*: Despite general free coverage, subtle differences in co-payments, reimbursement rates for long-acting methods (LARCs), or coverage of preparatory procedures can create varying levels of out-of-pocket costs across the UCS (public sector focus), SSS (mandated worker insurance), and CSMBS (government employee benefits). These cost differences may steer women in one scheme toward cheaper, short-acting methods and women in another toward more expensive, long-term options.
- *Service Accessibility and Network Structure*: The three schemes rely on distinct provider networks. CSMBS members typically access care through government hospitals with established specialty services, potentially making permanent methods (sterilization) easier to access. SSS members often use designated private or industrial clinics, which may favor dispensing hormonal or short-acting methods. UCS members rely heavily on primary care units, where access to insertion/removal procedures for LARCs might be logistically more complex.
- *Provider Incentives and Counseling*: The differing provider reimbursement and capitation [a payment model where a fixed, predetermined fee is paid to a healthcare provider or organization for each enrolled person, regardless of how many services are provided] models across the schemes can inadvertently influence provider counseling biases, potentially leading to varied recommendations for short-acting versus long-acting methods."

Systematically studying the relationship between the *specific health insurance type* and the *demand for specific modern contraceptive methods* is therefore essential. Such an analysis moves beyond the binary of 'insured/uninsured' to understand the sophisticated mechanisms through which public health systems impact women's autonomous health decisions [2].

Despite numerous studies on factors determining contraceptive use and access to health services in Thailand, existing research still presents certain limitations. First, most studies tend to focus on the overall picture of contraceptive use without delving into the nuanced differences in demand and behavior for "each specific type" of contraception (e.g., oral contraceptive pills, injectable contraceptives, implants, IUDs). Each option carries distinct costs, efficacies, usage methods, and potential side effects, all of which significantly influence women's decisions [8].

Second, while acknowledging the importance of health insurance systems, prior research lacks an in-depth, systematic analysis linking "the specific type of health insurance system a woman belongs to" with "her demand for and behavior in choosing each contraceptive option." Understanding how the coverage conditions and payment structures of distinct health insurance schemes influence different contraceptive method choices remains a significant research gap, particularly in Thailand's context of diverse health insurance systems with varying contraceptive benefits [16]. An analysis of demand for multiple contraceptive choices will provide insights into women's sequential preferences for various contraceptive methods under the constraints and benefits of their affiliated health insurance systems.

Third, this study leverages nationally representative survey data that encompasses a large and diverse sample population in Thailand. This ensures the reliability of the analysis and provides an accurate and systematic reflection of modern contraceptive demand among Thai women. Such national-level data are critical for examining complex economic factors, such as the influence of health insurance systems, which may differ across regions or population groups. Utilizing comprehensive data will facilitate the identification of key macro-level trends and driving factors.

The current study aims to address these research gaps by systematically analyzing the influence of health insurance types on the demand for and behavior in choosing modern contraceptive methods among Thai women, while controlling for relevant economic, social, and demographic factors. This analysis seeks to yield actionable insights for future public health policy and family planning strategies. The study is organized as follows: Sect. " Health insurance and contraceptive coverage in Thailand" describes Thailand's health insurance system and its theoretical connection to contraceptive demand. Sect. " Data" details the data and methodology used. Sect. " Econometric Results" presents the econometric results regarding the factors affecting contraceptive method selection. Sect. " Discussion" discusses these findings in the context of structural mechanisms and socioeconomic behaviors. Finally, Sect. " Conclusion and Policy Recommendations" offers concluding remarks and policy recommendations.

## Health insurance and contraceptive coverage in Thailand

Thailand has developed a health insurance system designed to provide comprehensive access to essential medical services for the majority of its population. This system comprises three primary types, each differing in target population, funding sources, and benefits. While all three aim to provide healthcare, their specific provisions, particularly regarding family planning and contraceptive services, vary. These variations significantly influence Thai women's decisions and service utilization behaviors.

The first system is the Universal Coverage Scheme (UCS), commonly known as the "Gold Card." This is Thailand's largest scheme, covering most of the population not eligible for other systems. Funded by the national budget, it focuses on providing comprehensive, essential basic health services free of charge at the point of service including pills, injections, and sterilization. Many family planning and contraceptive services—including pills, injections, implants, IUDs, and sterilization—are covered under this scheme. Crucially, the UCS is designed to be the fallback scheme for all Thai citizens who do not have entitlements under the other systems (SSS or CSMBS), thereby ensuring near-universal health coverage in the country [20].

The second system is the Social Security Scheme (SSS), which primarily covers private sector employees and voluntary self-employed participants. It is jointly funded by contributions from employees, employers, and the government. SSS benefits cover illness, childbirth, disability, and health promotion/disease prevention services, including various types of contraception comparable to those offered by the Gold Card. Insured individuals predominantly receive medical services from their designated hospitals [26].

The third system is the Civil Servant Medical Benefit Scheme (CSMBS), which provides benefits to civil servants, permanent government employees, and their families. Directly funded by the government, eligible individuals can access services at almost any public hospital and can claim medical expenses at a higher reimbursement rate. However, its family planning coverage is structured distinctly: the CSMBS directly covers only permanent sterilization costs. For temporary contraceptive methods (e.g., pills, injections, IUDs, implants), CSMBS members must utilize their parallel benefits under the National Health Security Office (NHSO), which manages the Universal Coverage Scheme (UCS). This necessity for members to switch schemes based on the type of service sought creates a structural access barrier that reinforces our finding that the specific scheme significantly influences method choice [20].

Table 1 would typically illustrate the differences among these health insurance systems, detailing their funding sources, direct benefit coverage for specific contraceptive types, and service access channels. These factors are crucial as they can directly influence women's out-of-pocket expenses, thereby affecting the ease of accessing different contraceptive methods. Understanding the dynamics of these systems is key to analyzing the demand for and choice of modern contraception in Thailand.

**Table 1:** Comparative Summary of Health Insurance Systems in Thailand and Contraceptive Coverage

Feature	Universal Coverage Scheme (UCS)	Social Security Scheme (SSS)	Civil Servant Medical Benefit Scheme (CSMBS)
Target Group	All Thai citizens without other entitlements	Private sector employees (Sect. 33), Voluntary insured (Sects. 39, 40)*	Civil servants, permanent government employees, and their family members
Funding Source	National budget (via NHSO)	Contributions from employees, employers, and government	National budget (via Comptroller General's Department)
Main Benefits	Comprehensive essential basic health services; largely no out-of-pocket costs	Sickness, maternity, disability, child welfare, old age, unemployment, death benefits	Medical treatment costs, medication, surgery at public hospitals (reimbursed based on actual cost/high rate)
Contraceptive Coverage	Covers contraceptive pills, injections, implants, IUDs, condoms, sterilization (no out-of-pocket costs at point of service for eligible individuals)	Covers contraception similar to UCS (as health promotion services are co-managed with NHSO)	Directly excludes all types of temporary contraception (pills, injections, implants, IUDs), except sterilization. However, eligible individuals can utilize NHSO benefits for some temporary contraceptive services
Service Access	Registered with primary care unit in the area; "30 Baht Universal Healthcare" policy for care anywhere	Selects registered primary healthcare facility; referral system in place	Access to almost any public hospital nationwide
Out-of-Pocket Costs	Very low (mostly no charge)	Low (free treatment at registered hospital)	None/Reimbursable (for covered items), but some temporary contraceptive services require using NHSO benefits

Source: Compiled and summarized by the researchers

^*^These sections refer to the categories of individuals covered under the Thai Social Security Scheme (SSS): Sect. 33 covers mandatory private sector employees; Sect. 39 covers individuals who voluntarily maintain coverage after leaving mandatory employment; and Sect. 40 covers self-employed and informal workers who enroll voluntarily for basic benefits

In particular, individuals covered by the Universal Health Coverage (UHC) scheme, which is popularly known in Thailand as the "30 Baht Scheme." This name references the nominal 30 Thai Baht (approximately US$1) co-payment that was initially required per outpatient visit when the scheme was introduced in 2001 (this was a per-visit fee, not a monthly insurance premium, and has since been waived for most services). UHC members can access nearly all types of contraceptive services under the Health Promotion and Disease Prevention (PP) program. This program targets all Thai citizens of reproductive age, from 8 to 59 years, 11 months, and 29 days, with parental consent required for those under 20. Covered services include oral contraceptive pills, emergency contraceptive pills, IUD insertion, contraceptive injections, condoms, and permanent sterilization.

Significantly, individuals with Civil Servant and Social Security rights, whose primary schemes may not cover all contraceptive methods, can still utilize the Health Promotion and Disease Prevention (PP) services provided by the National Health Security Office (NHSO). This is because PP benefits are universally offered to all Thai citizens regardless of their primary health insurance type, aiming to reduce health risk factors, lower morbidity and mortality rates, and ultimately yield long-term savings in healthcare expenses.

While the universal nature of the Health Promotion and Disease Prevention (PP) program ensures a baseline of free contraceptive access for all Thai citizens, we posit that the structural differences in the three primary schemes introduce distinct behavioral incentives that influence the choice of method. This mechanism goes beyond general financial access and encompasses three key areas. Firstly, Network Constraints and Convenience create a 'network lock-in' effect, particularly for SSS members, who are often restricted to their designated hospital. If that hospital has a limited family planning department or long wait times for specialized procedures like IUD insertion or sterilization, members may be subtly steered toward more readily available, short-acting methods like pills or injections, which require less specialized clinic time. Conversely, CSMBS members possess greater flexibility to choose facilities, potentially increasing their propensity for methods requiring specialized procedures, like permanent sterilization. Secondly, Provider Incentives and Method Promotion play a role, as the schemes use different financing and reimbursement models (e.g., capitation under UHC versus potential fee-for-service components under CSMBS). These models may inadvertently influence provider counseling and the active promotion of specific methods, leading to a subtle, systemic bias in recommendations toward methods that are logistically simpler or offer better institutional reimbursement. Finally, Perceived Financial Risk also contributes; although the PP benefit covers many methods, women enrolled in the schemes typically associated with higher income (CSMBS and SSS) may be more willing to absorb minor, non-reimbursable costs (e.g., for follow-up care or preferred brands) for long-term, highly effective methods, relative to women under the UHC.

Therefore, analyzing the demand for specific modern methods in the context of these three disparate schemes is crucial for identifying structural barriers and unintended consequences that persist, even under universal access.

Understanding which methods are preferred under which scheme will allow policymakers to align benefits and service delivery to maximize informed choice and promote the uptake of highly effective contraception across all population groups.

When comparing the efficacy of modern contraceptive methods, contraceptive implants demonstrate the highest effectiveness in preventing pregnancy, boasting a success rate of 99.9%. This is closely followed by male sterilization (99.9%) and female sterilization (99.5%). However, as sterilization is a permanent solution, it is only suitable for individuals definitively certain about not desiring future pregnancies. For those seeking the most cost-effective temporary contraception, oral contraceptive pills are a highly effective option (99.7% when used correctly) with a relatively low cost. They are ideal for individuals who can consistently adhere to a daily regimen. For those who find consistent medication use challenging or tend to forget methods requiring frequent reapplication, contraceptive patches or vaginal rings offer comparable efficacy (99.7%) and greater long-term convenience. Male condoms present another versatile option, suitable for all situations, particularly for irregular sexual activity or when protection against sexually transmitted infections is also required. They can also be used in conjunction with other methods to enhance effectiveness and minimize the risk of failure. In emergencies, such as after unprotected intercourse or condom breakage, emergency contraceptive pills are available. Although their efficacy is approximately 85%, they can significantly reduce the chance of pregnancy if administered within the stipulated timeframe [7, 21, 28]. 

As illustrated in Table 2, which compares contraceptive efficacy and cost, each method possesses distinct advantages and limitations. Therefore, the choice of contraception should account for lifestyle suitability, ease of use, desired duration of contraception, and acceptable cost. Fortunately, due to the support from the National Health Security Office (NHSO), which covers various contraceptive services, the public can more easily access effective contraceptive methods without substantial financial burden. Promoting a wide range of accessible contraceptive options for the public is a crucial strategy for fostering robust and sustainable sexual health.

**Table 2:** Comparison of Contraceptive Efficacy and Cost (Prices in Thai Baht)

Contraceptive method typical use	Suitable for	Efficacy (%)		Approximate price (Thai Baht)
		Typical Use (with potential for error)	Perfect Use (without error)	
Male Condom	Suitable for all occasions, particularly for infrequent sexual activity or as a backup when other methods fail. Also provides protection against sexually transmitted infections	85.0	98.0	50–135 (Contained 3 pieces)
Female Condom		79.0	95.0	150–300 (1 layer)
Oral Contraceptive Pills	Individuals seeking temporary contraception who demonstrate discipline in consistent daily use	92.0	99.7	50–500
Contraceptive Patch		92.0	99.7	400–600
Vaginal Ring		92.0	99.7	400–500
Emergency Contraceptive Pill	For emergency use only, following unprotected intercourse or contraceptive failure (e.g., condom breakage)	No data	85.0	50–80
Contraceptive Injection	Individuals seeking temporary contraception who prefer a non-daily regimen	97.0	99.7	300–915 (Excludes injection fee)
Intrauterine Device (IUD) (3–5 years)	Individuals seeking for more effective long-acting contraception	99.2	99.4	3,000–11,090
	Individuals tending to forget to take oral medication, apply patches, or receive injections			
	Individuals who have deficiencies in life skills pertaining to the use of other contraceptive methods			
Contraceptive Implant (3–5 years)	Couples who have completed childbearing but prefer a reversible, long-acting option over permanent sterilization	99.9	99.9	Government Hospitals: 2,500–4,000 Private Hospitals: 5,000–8,000
Male Sterilization (Permanent)	Individuals or couples who have completed childbearing	99.8	99.9	Government Hospitals: 500–3,00 Private Hospitals: 13,000–15,000

Source: Compiled and summarized by the researchers from Thailand's Ministry of Public Health. (Accessed at: https://multimedia.anamai.moph.go.th/infographics/which-birth-control-method-is-right-for-you/)

## Data

This study utilized data from the Multiple Indicator Cluster Survey (MICS) 2022, a nationally representative survey supported by UNICEF in collaboration with the Royal Thai Government. MICS aims to collect comprehensive data on the situation of children and women at the national level, facilitating the creation of international indicators for cross-country comparisons, informing policy development, and evaluating progress toward the Sustainable Development Goals (SDGs) and other international agreements.

The survey collected data nationwide, stratified by urban and rural areas and across five regions (Bangkok, Central, Northern, Northeastern, and Southern), employing multi-stage stratified cluster sampling. From an initial sample of 35,540 households, this study focused on questionnaires administered to reproductive-aged women (15–49 years old). A total of 21,663 women were eligible for interview, with 21,089 successfully interviewed. Of these, 10,922 women (53.05% of the sample) were currently using methods to space out or avoid pregnancy.

The first dependent variable was "Contraceptive Use Status," captured by a binary (yes/no) response to the question: "Are you or your partner currently using a method to space out or avoid pregnancy?" This comprehensive filter includes both modern and traditional contraceptive methods, which is essential for accurately setting the baseline for subsequent comparative analysis. Disaggregating contraceptive use by socioeconomic factors (Table 3) revealed several patterns. Demand for contraception generally increased with age: only 24.8% among adolescents (15–24 years), rising to 54.2% (25–29 years), peaking at 65.2% (35–39 years), and then slightly declining after age 40. Contraceptive demand was highest among married women (76.9%) and those residing in non-municipal areas (57%) compared to municipal areas (49.5%). Muslim women exhibited the lowest demand (39.6%). This lower rate among some Muslim groups stems from diverse religious interpretations, cultural values emphasizing childbearing, social pressures, and limited access to information or services, rather than direct religious prohibition. Initial analysis across the three health insurance schemes showed minimal differences in overall contraceptive use (approximately 52–54% of insured individuals used contraception across types).

**Table 3:** Percentage and Number of Thai Reproductive-Age Women Using Contraception

Independent Variables	Currently Using Contraception (%)	Modern Contraception (%)			Traditional Contraception (%)	Number of Contraceptive Users	Total Sample Size
		Permanent (Female/Male Sterilization)	Hormonal (Injection, Pill, Implant)	Device/Barrier (Condom, IUD, Diaphragm)			
Administrative Area							
Non-Municipal	57.0	34.3	58.1	2.6	5.0	5,558	9,746
Municipal	49.5	34.6	55.2	3.4	6.9	5,361	10,842
Region							
Bangkok Metropolitan	42.7	25.1	63.0	5.5	6.5	915	2,143
Central	52.9	33.2	56.1	4.8	5.9	1,641	3,105
Northern	62.1	35.5	58.8	1.9	3.8	2,021	3,253
Northeastern	57.5	45.4	50	1.9	2.7	3,195	5,563
Southern	48.2	25.0	60.5	3.0	10.6	3,147	6,524
Religion							
Christian	59.2	30.0	67.6	0.7	0.7	142	240
Buddhist	55.6	37.3	54.8	3.0	4.9	9,449	16,999
Muslim	39.6	14.8	68.4	2.7	14.1	1,319	3,328
Age Group							
15–24	24.8	5.0	86.1	3.7	5.3	1,097	4,434
25–29	54.2	19.5	69.0	3.7	7.9	1,775	3,274
30–34	62.5	31.6	59.3	2.9	6.2	2,149	3,440
35–39	65.2	39.6	51.0	3.3	6.0	2,251	3,454
40–44	62.8	45.9	45.7	2.5	6.0	1,960	3,123
45–49	58.9	52.8	41.4	1.9	3.9	1,687	2,863
Education Level							
Primary School	65.6	39.9	54.9	1.0	4.2	2,297	3,504
Lower Secondary School	62.1	33.3	60.3	2.0	4.5	2,413	3,888
Upper Secondary/Vocational	48.8	32.8	58	3.6	5.7	3,445	7,060
Bachelor's Degree or Higher	42.8	34	50.9	5.3	9.8	2,336	5,457
Wealth Index Quintile							
Poorest	59.1	32.6	62.9	1.1	3.4	2,198	3,720
Poor	52.1	30.7	62.1	2.2	5.1	2,261	4,338
Middle	51.8	33.9	57.7	3.0	6.1	2,406	4,647
Rich	51.8	37.3	52.6	3.5	6.6	2,256	4,354
Richest	51	39.5	45.8	5.6	9.1	1,798	3,529
Marital Status							
Never Married, Not Cohabiting	0.8	18.4	57.9	15.8	7.9	38	5,123
Legally Married	76.9	33.5	57.5	3.0	6.1	10,459	13,612
Widowed, Divorced, Separated	22.8	59.2	36.7	1.4	2.6	422	1,853
Age at First Cohabitation							
≤ 17 years	65.8	34.0	62.2	1.9	1.9	156	237
> 17 years	52.9	34.5	56.6	3.0	6.0	10,763	20,351
Health Insurance Scheme							
Social Security Scheme (SSS)	54.5	29.8	60.2	4.0	6.0	2,342	4,303
Civil Servant Medical Benefit Scheme (CSMBS)	53.5	42.9	43.5	5.6	8.0	699	1,307
Universal Coverage Scheme (UCS)	52.7	35.5	56.5	2.4	5.6	7,277	13,809
Total (Average)	53.1	34.5	56.7	3.0	5.9	10,919	20,588

Source: Calculated by the researchers based on raw data from the Multiple Indicator Cluster Survey (MICS) 2022

The analysis focuses on Buddhist, Muslim, and Christian populations. Other religious groups (e.g., Hindu, Sikh) represented less than 1% of the total sample and were excluded from the regression analysis to ensure statistical robustness and avoid small cell size bias

Interestingly, women with higher educational attainment had lower overall contraceptive demand. Similarly, women in the wealthiest income quintile demonstrated lower demand (51%) compared to poorer women. This finding deviates from general theoretical expectations, which typically hold that higher socioeconomic status and education correlate with increased contraceptive uptake due to better knowledge, improved service access, and greater autonomy in decision-making. This apparent paradox is hypothesized to stem not from a lack of knowledge or access, but from a behavioral substitution observed among affluent and highly educated women: they tend to avoid highly effective hormonal methods due to increased sensitivity to perceived side effects (e.g., weight gain, mood changes). Instead, this demographic often opts for natural or traditional methods (e.g., withdrawal, rhythm method), leveraging their knowledge and personal discipline, or relies on abstinence or barrier methods outside the "current contraceptive use" definition [9, 21, 25]. This pattern suggests that, for this specific socioeconomic group, the decision is less about overcoming financial barriers and more about making highly informed, personal choices regarding method side effects, which the subsequent Multinomial Logit analysis is designed to confirm.

The second dependent variable was "Type of Contraceptive Method Chosen." Respondents using contraception specified their method, which researchers categorized into four main types: 1) Permanent Methods (female and male sterilization); 2) Hormonal Methods (injections, pills, implants); 3) Device/Barrier Methods (condoms, IUDs, diaphragms); and 4) Natural Methods (rhythm method, withdrawal, foams, lubricants, etc.). The first three are considered modern methods, while the last comprises traditional techniques.

Table 3 shows the most popular choice was Hormonal Methods (56.7%), followed by Permanent Methods (34.5%), Traditional Methods (5.9%), and lastly, Device/Barrier Methods (3%). Overall, 94.06% of reproductive-aged women using contraception in Thailand opted for modern methods.

Conversely, traditional or natural contraceptive methods showed a higher proportion among Muslim women (14.10%) and those residing in the Southern region (10.55%), which has a significant Muslim population. This aligns with Islamic teachings that often discourage permanent contraception, primarily due to religious doctrines emphasizing childbearing and procreation, coupled with a belief in Allah's provision of sustenance ("Rizq"), which can lead to permanent contraception being perceived as a lack of divine trust [1].

Notably, natural contraceptive use was also relatively high among highly educated (Bachelor's/Master's/PhD) women (9.76%) and the wealthiest women (9.12%), particularly those aged 17 and above (6.01%). This phenomenon—where some highly educated and affluent women prefer traditional/natural methods—can be attributed to several factors. Research suggests that highly educated women are often more health-conscious and have greater access to health information, leading some to avoid hormone-based contraceptives due to concerns about potential side effects like mood swings, weight gain, or long-term health risks such as blood clots [12, 15].

**Fig. 1:**
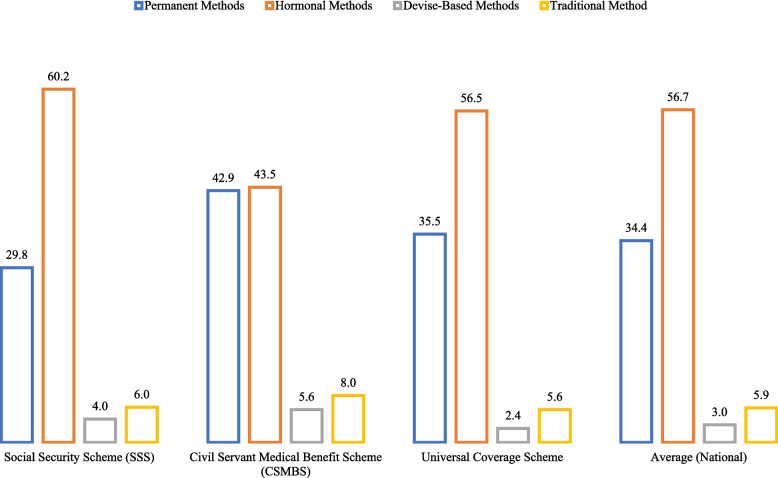
Proportion of Contraceptive Choices by Health Insurance Scheme Type. Source: Calculated by the researchers, based on raw data from the Multiple Indicator Cluster Survey (MICS) 2022

As depicted in Fig. 1, contraceptive method choices vary significantly across Thailand's different health insurance schemes when compared to the national average. Nationally, Hormonal Methods (56.65%) and Permanent Methods (34.5%) are the most dominant choices, with Device-Based Methods (3%) and Traditional (5.9%) methods being far less common. Women covered by the Universal Coverage Scheme (UCS), the largest group, demonstrate choices that closely mirror this national average. However, distinct preferences emerge in the other schemes: women under the Social Security Scheme (SSS) show a notably higher propensity for Hormonal Methods (60.2%). In sharp contrast, members of the Civil Servant Medical Benefit Scheme (CSMBS) exhibit a high and nearly equal preference for Permanent Methods (42.9%) and Hormonal Methods (43.1%), alongside a significantly higher preference for Device-Based Methods (5.6%) than any other group. These initial observations suggest that the specific structure of a woman's health insurance scheme influences the demand for different contraceptive types.

However, these initial analyses alone are not definitive as they lack systematic statistical correlation. Employing appropriate statistical methods will enable clearer and more precise identification and explanation of relationships between variables, while also controlling for other factors influencing contraceptive use. The subsequent section will address these limitations through econometric modeling.

## Econometric results

This econometric analysis is structured into two main parts. First, we employ a Probit Regression model to estimate the probability (expressed as marginal effects) of overall contraceptive use among reproductive-age women (15–49 years old) in Thailand. Second, we employ a Multinomial Logistic Regression model to specifically analyze the determinants of choosing between Modern Methods (Permanent, Hormonal, Device-Based) versus Traditional Methods.

The analysis includes four specifications. Model 1 estimates the probability of overall contraceptive use with socioeconomic control variables. Model 2 adds marital status and age at first marriage. Model 3 incorporates the three health insurance schemes (SSS, CSMBS, UCS). Model 4 estimates the probability of choosing modern contraceptive methods specifically.

It is important to note that a direct monetary price variable for contraceptive methods was excluded from the estimation models. This exclusion is due to two primary factors: first, the MICS is a household survey and does not capture market price data faced by respondents; second, in the Thai context, contraceptive services are largely subsidized under the national health schemes (effectively zero or fixed low cost). Therefore, the 'Health Insurance Scheme' variable serves as the primary proxy for the economic cost of access in this analysis. Furthermore, prior to estimation, Variance Inflation Factor (VIF) tests were conducted to detect potential multicollinearity among the independent variables. All VIF values were found to be below 2.5, indicating that multicollinearity is not a significant concern in these models.

### Determinants of overall contraceptive use (probit model)

Table 4 presents the estimation results from the Probit models. The results indicate a significant disparity based on residential area; individuals residing in municipal areas are approximately 4.8 percentage points less likely to use contraception compared to those in non-municipal areas. Regionally, the Northern region showed the highest probability of contraceptive adoption (9% to 15.2% higher compared to Bangkok), followed by the Northeastern (2.5–11.2%), Central (4.8–6.3%), and Southern (2.8–8%) regions.

**Table 4:** Probit Model Estimation of Contraceptive Demand (Marginal Effects)

Independent Variable	Contraceptive Use					Model 4 (Modern Contraceptive Use)	
	Model 1		Model 2		Model 3		
Residential Area (Ref: Non-Municipal)							
Municipal	−0.048***		−0.012		−0.013	−0.014***	
	*(0.008)*		*(0.010)*		*(0.010)*	*(0.004)*	
Region (Ref: Bangkok Metropolitan)							
Central	0.063***		0.048**		0.044**	0.002	
	*(0.015)*		*(0.020)*		*(0.021)*	*(0.009)*	
Northern	0.152***		0.091***		0.090***	0.008	
	*(0.015)*		*(0.020)*		*(0.022)*	*(0.008)*	
Northeastern	0.112***		0.048***		0.044**	0.025***	
	*(0.014)*		*(0.018)*		*(0.019)*	*(0.007)*	
Southern	0.080***		−0.020		−0.023	−0.028***	
	*(0.015)*		*(0.018)*		*(0.020)*	*(0.010)*	
Religion (Ref: Christian)							
Buddhist	0.098**		0.026		0.018	−0.035*	
	*(0.038)*		*(0.047)*		*(0.049)*	*(0.020)*	
Muslim	−0.066		−0.174***		−0.181***	−0.137	
	*(0.040)*		*(0.043)*		*(0.045)*	*(0.089)*	
Age (years)	0.131***		0.024***		0.024***	0.001	
	*(0.003)*		*(0.005)*		*(0.005)*	*(0.002)*	
Age^2^	−0.002***		−0.001***		−0.001***	−0.001	
	*(0.001)*		*(0.001)*		*(0.0001*	*(0.001)*	
Education (Ref: Primary School)							
Lower Secondary School	0.007		0.012		0.005	−0.000	
	*(0.013)*		*(0.014)*		*(0.015)*	*(0.007)*	
Upper Secondary/Vocational	−0.104***		−0.029**		−0.034**	−0.010	
	*(0.012)*		*(0.014)*		*(0.014)*	*(0.007)*	
Bachelor's Degree or Higher	−0.275***		−0.092***		−0.095***	−0.032***	
	*(0.012)*		*(0.015)*		*(0.017)*	*(0.010)*	
Wealth Index (Ref: Poorest)							
Poor (Q2)	−0.024*		0.002		−0.000	−0.001	
	*(0.013)*		*(0.015)*		*(0.015)*	*(0.008)*	
Middle (Q3)	−0.012		−0.013		−0.018	−0.009	
	*(0.013)*		*(0.015)*		*(0.015)*	*(0.008)*	
Rich (Q4)	0.008		−0.012		−0.016	−0.013	
	*(0.013)*		*(0.016)*		*(0.016)*	*(0.009)*	
Richest (Q5)	0.039***		−0.010		−0.016	−0.038***	
	*(0.015)*		*(0.018)*		*(0.018)*	*(0.012)*	
Marital Status (Ref: Never Married, Not Cohabiting)							
Legally Married	-		0.801***		0.803***	0.025	
	*-*		*(0.005)*		*(0.005)*	*(0.041)*	
Widowed, Divorced, Separated	-		0.535***		0.533***	0.035***	
	*-*		*(0.017)*		*(0.017)*	*(0.013)*	
Age at First Cohabitation (Ref: Over 17 years)							
17 years or younger	-		−0.028		−0.031	0.028**	
	*-*		*(0.037)*		*(0.037)*	*(0.012)*	
Health Insurance Scheme (Ref: Universal Coverage Scheme (UCS))							
Social Security Scheme (SSS)	-		-		0.017	0.007	
	*-*		*-*		*(0.013)*	*(0.005)*	
Civil Servant Medical Benefit Scheme (CSMBS)	-		-		0.015	0.008	
	*-*		*-*		*(0.020)*	*(0.007)*	
Observations	19,889		19,889		18,959	10,026	
Pseudo R-squared	0.141		0.425		0.426	0.0770	

Standard errors in parentheses. Raw data from the Multiple Indicator Cluster Survey (MICS) 2022

^***^p < 0.01, **p < 0.05, *p < 0.1

Regarding individual factors, age was a significant determinant, with the probability of use increasing by 2.4–13.1 percentage points for every one-year increase. Religion also played a role: Buddhist women were 9.8 percentage points more likely to choose contraception compared to Christian women, while Muslim women exhibited the lowest demand. Economic status showed a positive correlation, as women in the wealthiest quintile were approximately 3.9 percentage points more likely to use contraception than those in the poorest quintile.

However, education level presented a negative correlation: women with a Bachelor's degree or higher were approximately 2.75–3.2 percentage points less likely to use contraception compared to those with a primary education. This pattern persisted when analyzing the demand for modern contraceptive methods specifically; wealthy, highly educated women residing in municipal areas had a decreased likelihood of using modern contraception (falling by 1.4 percentage points).

Crucially, in the aggregate Probit models (Model 3 and Model 4), the specific health insurance scheme type did not show a statistically significant relationship with the overall demand for contraception. This suggests that while insurance facilitates access, the distinction between schemes does not drive the binary decision of whether to use contraception or not. To understand the impact of insurance on *method choice*, we proceed to the Multinomial Logistic Regression.

### Determinants of method choice (multinomial logit model)

Table 5 presents the Odds Ratios (OR) from the Multinomial Logistic Regression, categorizing choices into Permanent, Hormonal, and Device-Based methods, with Traditional methods as the reference group.

**Table 5:** Estimated Odds Ratios from Multinomial Logistic Regression for Contraceptive Method Choices (Reference: Traditional Methods such as Rhythm Method, Withdrawal, etc.)

Independent Variables	1	2	3	4	5	6
	Permanent Methods (Female, Male Sterilization)	Device-Based Methods (Condom, IUD, Diaphragm)	Hormonal Methods (Injection, Pill, Implant)	Permanent Methods (Female, Male Sterilization)	Device-Based Methods (Condom, IUD, Diaphragm)	Hormonal Methods (Injection, Pill, Implant)
	Reference: Traditional Methods (Rhythm Method, Withdrawal, etc.)					
Administrative Area (Ref: Non-Municipal Area)						
Municipal Area	0.819*	0.741*	0.750**	0.806*	0.741	0.734**
	*(0.078)*	*(0.112)*	*(0.068)*	*(0.080)*	*(0.116)*	*(0.069)*
Region (Ref: Bangkok Metropolitan)						
Central	1.349	0.865	0.825	1.438	0.882	0.934
	*(0.264)*	*(0.225)*	*(0.152)*	*(0.295)*	*(0.239)*	*(0.182)*
Northern	1.795**	0.503*	0.986	1.766**	0.525*	1.129
	*(0.366)*	*(0.148)*	*(0.191)*	*(0.382)*	*(0.161)*	*(0.233)*
Northeastern	3.230***	0.718	1.253	3.300***	0.741	1.519*
	*(0.631)*	*(0.194)*	*(0.234)*	*(0.690)*	*(0.213)*	*(0.304)*
Southern	0.828	0.372***	0.475***	0.811	0.356***	0.540***
	*(0.149)*	*(0.095)*	*(0.080)*	*(0.155)*	*(0.096)*	*(0.096)*
Religion (Ref: Christian)						
Buddhist	0.303	0.513	0.225	0.347	0.542	0.257
	*(0.310)*	*(0.731)*	*(0.228)*	*(0.355)*	*(0.773)*	*(0.260)*
Muslim	0.073*	0.250	0.150	0.077*	0.261	0.166
	*(0.076)*	*(0.361)*	*(0.153)*	*(0.080)*	*(0.376)*	*(0.170)*
Age						
Age (years)	1.451***	0.907	0.952	1.507***	0.935	0.968
	*(0.078)*	*(0.069)*	*(0.046)*	*(0.084)*	*(0.073)*	*(0.048)*
Age^2^	0.996***	1.001	1.001	0.995***	1.001	1.000
	*(0.001)*	*(0.001)*	*(0.001)*	*(0.001)*	*(0.001)*	*(0.001)*
Education (Ref: Primary Education)						
Lower Secondary Education	1.111	1.523	0.989	1.080	1.528	0.966
	*(0.176)*	*(0.453)*	*(0.151)*	*(0.178)*	*(0.472)*	*(0.153)*
Upper Secondary, Vocational	0.849	2.191**	0.829	0.825	2.147**	0.784
	*(0.125)*	*(0.597)*	*(0.117)*	*(0.127)*	*(0.611)*	*(0.115)*
Bachelor's Degree or Higher	0.507***	2.002*	0.579***	0.525***	1.874*	0.560***
	*(0.081)*	*(0.577)*	*(0.088)*	*(0.092)*	*(0.584)*	*(0.094)*
Wealth Index (Ref: Poorest)						
Poor	0.874	1.365	0.894	0.929	1.390	0.963
	*(0.150)*	*(0.417)*	*(0.147)*	*(0.166)*	*(0.433)*	*(0.165)*
Middle	0.898	1.459	0.773	0.885	1.393	0.772
	*(0.151)*	*(0.433)*	*(0.125)*	*(0.153)*	*(0.417)*	*(0.128)*
Rich	0.938	1.387	0.676*	0.918	1.289	0.662*
	*(0.163)*	*(0.418)*	*(0.113)*	*(0.164)*	*(0.393)*	*(0.113)*
Richest	0.700	1.344	0.453***	0.642*	1.086	0.422***
	*(0.130)*	*(0.422)*	*(0.081)*	*(0.124)*	*(0.348)*	*(0.078)*
Marital Status (Ref: Single, Not Cohabiting)						
Legally Married	1.796	0.410	1.707	1.757	0.411	1.919
	*(1.290)*	*(0.296)*	*(1.073)*	*(1.273)*	*(0.298)*	*(1.219)*
Widowed, Divorced, Separated	5.689*	0.423	1.975	6.230*	0.432	2.588
	*(4.453)*	*(0.372)*	*(1.385)*	*(4.994)*	*(0.394)*	*(1.866)*
Cohabiting with Partner Aged < 17 years (Ref: Aged > 17 years)	-	-	-	2.582	2.145	2.410
	*-*	*-*	*-*	*(1.573)*	*(1.776)*	*(1.430)*
Health Insurance Coverage (Ref: Universal Coverage Scheme (UCS))						
Social Security Scheme (SSS)	-	-	-	0.996	1.142	1.325*
	-	-	-	*(0.125)*	*(0.210)*	*(0.159)*
Civil Servant Medical Benefit Scheme (CSMBS)	-	-	-	1.196	1.734*	1.178
	-	-	-	*(0.211)*	*(0.4563)*	*(0.2108)*
Sample Size	10,483			10,026		
Pseudo R^2^	0.103			0.105		

Standard errors in parentheses. Raw data from the Multiple Indicator Cluster Survey (MICS) 2022

^***^p < 0.01, **p < 0.05, *p < 0.1

The analysis reveals a statistically significant association between a woman’s health insurance scheme and her specific contraceptive method choice. Individuals with Social Security Scheme (SSS) rights demonstrate a statistically significant 32.5% increased likelihood of choosing Hormonal Methods (pills, injections, implants) compared to Traditional methods, relative to the UCS reference group (OR = 1.325). Conversely, members of the Civil Servant Medical Benefit Scheme (CSMBS) are approximately 73.4% more likely to choose Device-Based Methods (Condom, IUD, Diaphragm) than those with UCS (OR = 1.734).

Geographically, the results corroborate the Probit findings but with greater nuance. Women in the Northeastern and Northern regions are significantly more likely to opt for Permanent Contraception (OR = 3.3 and 1.766, respectively) compared to those in Bangkok. However, women in municipal areas are generally 19.4% to 26.6% less likely to choose any modern method compared to traditional methods than their non-municipal counterparts.

Socioeconomic factors also showed distinct patterns in method selection. Women with a bachelor's degree or higher are approximately 47.5% less likely to choose permanent methods and 44% less likely to choose hormonal methods, but 87.4% more likely to select device-based methods compared to the primary education group. Similarly, women in the wealthiest quintile are significantly less likely to choose permanent sterilization (OR = 0.642) and hormonal methods (OR = 0.422).

## Discussion

The findings from this study highlight complex structural and behavioral mechanisms driving contraceptive choices in Thailand. This section discusses the three key themes emerging from the econometric results: the urban–rural paradox, the structural influence of health insurance schemes, and the socioeconomic "substitution effect."

### The urban–rural paradox: supply-side interventions

Contrary to global trends where urbanization typically correlates with higher contraceptive access, our study found that women in non-municipal (rural) areas and poorer regions (North, Northeast) exhibit higher contraceptive demand than those in municipal areas and Bangkok. This "Thai Paradox" can be attributed to the country's unique public health history. Thailand’s family planning program has been successful largely due to decentralized, proactive service delivery in rural areas through Sub-district Health Promoting Hospitals and the Village Health Volunteers (VHV) network. These mechanisms ensure that contraceptive services are brought directly to the community. In contrast, urban residents rely more on reactive care (travelling to hospitals) or purchasing contraception at pharmacies, which may lead to lower reported usage in standard surveys or a reliance on less effective methods. This aligns with findings from Indonesia by Sujarwoto et al. [19], observing that in rural contexts, national health insurance coverage significantly promotes uptake among women receiving direct governmental services.

### Health insurance structure and method choice mechanisms

The most novel finding of this study is that the *structure* of health insurance determines the *type* of contraceptive chosen. While overall coverage did not differ significantly across schemes, the Multinomial Logit results reveal distinct preferences: SSS members favor hormonal methods, while CSMBS members favor device-based methods.

We hypothesize that these preferences are driven by provider incentives and network constraints. The SSS operates on a capitation payment model, where hospitals receive a fixed fee per enrollee. This creates a financial incentive for providers to dispense cost-effective, time-efficient hormonal methods (pills/injections) rather than performing labor-intensive procedures like IUD insertion. Additionally, SSS members are often "locked in" to a specific hospital, making it logistically difficult to access specialized procedures if their designated facility is congested.

In contrast, the CSMBS operates on a fee-for-service model with a more flexible provider network. This allows civil servants to access specialized providers for medical devices or sterilization without the capitation constraints. Furthermore, the broader reimbursement policies of the CSMBS likely facilitate access to higher-quality barrier methods or IUDs, explaining the 73.4% higher likelihood of choosing device-based methods. This confirms that "universal coverage" does not imply "uniform access," as the payment mechanism significantly shapes the menu of options effectively available to women.

### Socioeconomic status and the quality-quantity trade-off

Our analysis revealed a counter-intuitive pattern where highly educated and wealthy women are *less* likely to use permanent or hormonal contraception and more likely to rely on natural or device-based methods. This phenomenon can be explained by a "substitution effect" driven by health literacy and side-effect concerns.

Research suggests that affluent, educated women are often more sensitive to the potential side effects of hormonal contraceptives (e.g., weight gain, mood changes, blood clot risks). Consequently, they substitute these highly effective clinical methods with natural methods (relying on personal discipline) or non-hormonal barrier methods (Device-Based), as confirmed by their high Odds Ratio for the latter category. This parallels findings in Western contexts where health-conscious women increasingly question hormonal interventions [25].

Additionally, cultural factors play a significant role. Muslim women, particularly in the Southern region, showed significantly lower uptake of permanent and hormonal methods. This aligns with religious interpretations regarding "Rizq" (divine provision) and the emphasis on procreation, leading to a preference for spacing births rather than permanently stopping them. These findings underscore that contraceptive demand is not merely a function of cost, but a complex interplay of structural access and personal values.

## Conclusion and policy recommendations

### Conclusion

This research aimed to analyze how Thailand’s diverse health insurance landscape—comprising the Universal Coverage Scheme (UCS), Social Security Scheme (SSS), and Civil Servant Medical Benefit Scheme (CSMBS)—influences modern contraceptive choices among reproductive-age women. Our approach intentionally moved beyond the simple insured/uninsured binary to isolate the structural impact of scheme differences, independent of standard socio-demographic factors.

Utilizing a multinomial logistic regression model built upon data from the 2022 Multiple Indicator Cluster Survey (MICS), the study confirms a statistically significant and distinct association between scheme membership and method choice. The key findings reveal a bifurcation in preferences across schemes when comparing modern method selection against the reference group of Traditional Methods.

Specifically, members of the Social Security Scheme (SSS) show a statistically significant higher propensity to select Hormonal Contraception (including Injections, Pills, and Implants). This preference is likely driven by the scheme's capitation payment model, which incentivizes providers to offer lower-cost, time-efficient hormonal methods rather than labor-intensive procedures. Additionally, for working women under the SSS, the convenience of hormonal methods may outweigh the logistical challenges of scheduling invasive procedures within a restrictive provider network.

Conversely, Civil Servant Medical Benefit Scheme (CSMBS) members are significantly more likely to opt for Device-Based Methods (Condoms, IUDs, and Diaphragms) compared to traditional methods. This distinct preference underscores the influence of the CSMBS’s flexible "Fee-for-Service" structure, which allows members to access specialized providers for medical devices (such as IUDs) or choose non-hormonal barrier methods. This aligns with the demographic profile of civil servants—often highly educated women—who may prioritize avoiding hormonal side effects, a choice facilitated by their scheme’s comprehensive reimbursement for medical devices.

These results constitute the core contribution of this study: the specific scheme of access and its underlying benefit structure significantly influences women's disparate contraceptive choices. This strongly suggests that different service delivery networks, provider incentives, and administrative barriers inherent to each scheme inadvertently steer women toward certain methods. Furthermore, the study confirmed that individual factors, such as religious beliefs (associated with lower permanent method uptake among Muslim women) and socioeconomic status (associated with higher non-hormonal method use among affluent, educated women due to side-effect concerns), remain crucial determinants.

In summary, merely guaranteeing general health rights or overall contraceptive access is insufficient. Public health policy must now pivot from focusing on the quantity of access to fundamentally improving the quality and structural alignment of contraceptive services within each distinct health insurance system to ensure comprehensiveness, flexibility, and responsiveness to the unique needs of diverse population groups.

### Policy recommendations

Based directly on the finding that scheme-specific structural features significantly influence contraceptive choice, the following policy recommendations are crucial for promoting appropriate and sustainable access to effective contraceptive methods:

First, policy must harmonize provider payment mechanisms to eliminate financial disincentives for long-acting methods. Our findings indicate that members of the Social Security Scheme (SSS) are significantly more likely to utilize Hormonal Methods (pills/injections) compared to traditional methods. This pattern suggests that the SSS capitation payment model may inadvertently incentivize providers to favor low-cost, quick-dispensing methods over labor-intensive procedures like IUD insertions. In contrast, the Civil Servant Medical Benefit Scheme (CSMBS), which operates on a fee-for-service basis, shows a distinct preference for Device-Based methods. To ensure that SSS members are not structurally steered away from clinical methods due to provider cost-containment strategies, policymakers should consider introducing specific "add-on" fee-for-service reimbursements for LARC insertion and removal within the SSS, similar to the flexibility observed in the CSMBS. This would ensure that a woman’s choice is driven by her health needs rather than her hospital’s financial constraints.

Second, administrative barriers within "locked-in" provider networks must be reduced to facilitate access to procedural contraception. While the "Device-Based" category includes barrier methods (condoms), the significant difference in method selection between the flexible CSMBS and the restrictive SSS suggests that network rigidity impacts choice. SSS members, who are restricted to a single hospital, may face logistical friction—such as long wait times or limited clinic hours—that makes scheduling a clinical procedure (like IUD insertion or sterilization) difficult compared to picking up pills. Policy should therefore mandate a rigorous review of referral protocols. If a designated SSS hospital cannot provide timely access to procedural contraception, the system must allow for seamless, penalty-free referrals to other public or private facilities, mirroring the "open access" convenience that CSMBS members currently exercise to access their preferred methods.

Finally, contraceptive counseling must be tailored to address the specific concerns of diverse socioeconomic groups. Our study confirms that highly educated and affluent women are less likely to choose hormonal methods, likely due to concerns regarding side effects. Furthermore, Muslim women demonstrate a distinct preference pattern, avoiding permanent methods. Therefore, a "one-size-fits-all" counseling approach is insufficient. Public health programs must upgrade counseling protocols to include: (1) proactive, evidence-based discussions on non-hormonal options (such as copper IUDs) for health-conscious, educated women who might otherwise default to less effective natural methods; and (2) culturally sensitive family planning education for religious communities. This targeted approach is vital to empowering all women to make fully informed choices that align with their lifestyle, beliefs, and health goals.

### Limitations and future research

This study has certain limitations that can inform future research. First and foremost, the cross-sectional nature of the MICS 2022 data limits our ability to establish definitive causal relationships. While we demonstrate a strong and significant *association* between a woman’s insurance scheme and her contraceptive method choice, we cannot rule out potential self-selection. For instance, women who already prefer long-acting methods might be more likely to work in the formal sector, which in turn enrolls them in the SSS. Future research using longitudinal panel data would be necessary to track women's choices over time, potentially as they move between schemes, to provide stronger evidence of a causal link.

Second, while our analysis proposes that structural factors like "network lock-in" (SSS) and "provider incentives" (CSMBS) are the primary drivers of our findings, the MICS dataset does not directly measure these mechanisms. The survey captures the *patient's* choice but not the *provider's* specific reimbursement rates or the logistical barriers (e.g., clinic wait times, referral complexity) a patient may have faced. This limitation underscores the need for future qualitative research. In-depth interviews with both patients *and* providers within each scheme are essential to confirm whether these hypothesized barriers and incentives are indeed the primary factors influencing decisions.

Third, our analysis is focused on the demand side (patient choice) and does not incorporate granular supply-side data. Future studies could integrate this MICS data with geographical or administrative data, such as the number of LARC-certified providers per SSS-registered hospital or the density of medical personnel in specific regions. This would help identify how service *availability* interacts with insurance scheme rules to shape outcomes, leading to more targeted policy interventions.

Finally, this study does not conduct a cost-effectiveness analysis of each contraceptive method under the different health insurance schemes. Such an analysis would provide valuable information for policymakers regarding the optimal allocation of public health budgets and resources to maximize both health outcomes and women's reproductive autonomy.

## Data Availability

No datasets were generated or analysed during the current study.
